# Mitochondrial Heteroplasmy Shifting as a Potential Biomarker of Cancer Progression

**DOI:** 10.3390/ijms22147369

**Published:** 2021-07-09

**Authors:** Carlos Jhovani Pérez-Amado, Amellalli Bazan-Cordoba, Alfredo Hidalgo-Miranda, Silvia Jiménez-Morales

**Affiliations:** 1 Laboratorio de Genómica del Cáncer, Instituto Nacional de Medicina Genómica, Mexico City 14610, Mexico; jhamado24@gmail.com (C.J.P.-A.); amellallibc@gmail.com (A.B.-C.); ahidalgo@inmegen.gob.mx (A.H.-M.); 2 Programa de Maestría y Doctorado, Posgrado en Ciencias Bioquímicas, Universidad Nacional Autónoma de México, Mexico City 04510, Mexico

**Keywords:** heteroplasmy, heteroplasmy shifting, mitochondrial DNA, mitochondrial mutations, cancer

## Abstract

Cancer is a serious health problem with a high mortality rate worldwide. Given the relevance of mitochondria in numerous physiological and pathological mechanisms, such as adenosine triphosphate (ATP) synthesis, apoptosis, metabolism, cancer progression and drug resistance, mitochondrial genome (mtDNA) analysis has become of great interest in the study of human diseases, including cancer. To date, a high number of variants and mutations have been identified in different types of tumors, which coexist with normal alleles, a phenomenon named heteroplasmy. This mechanism is considered an intermediate state between the fixation or elimination of the acquired mutations. It is suggested that mutations, which confer adaptive advantages to tumor growth and invasion, are enriched in malignant cells. Notably, many recent studies have reported a heteroplasmy-shifting phenomenon as a potential shaper in tumor progression and treatment response, and we suggest that each cancer type also has a unique mitochondrial heteroplasmy-shifting profile. So far, a plethora of data evidencing correlations among heteroplasmy and cancer-related phenotypes are available, but still, not authentic demonstrations, and whether the heteroplasmy or the variation in mtDNA copy number (mtCNV) in cancer are cause or consequence remained unknown. Further studies are needed to support these findings and decipher their clinical implications and impact in the field of drug discovery aimed at treating human cancer.

## 1. Introduction

Despite the growing understanding of cancer biology and the constant effort to develop more effective diagnostic, follow-up and therapeutic strategies, these diseases still have important socio–economic implications worldwide [1]. To identify new diagnostic and prognostic biomarkers and even new potential molecular targets, current research focuses on the nuclear genome by detecting mutations and on gene expression and epigenomic signatures [2,3,4,5]; however, given the number of biological processes in which mitochondria participate (energy synthesis, metabolism, apoptosis and signaling pathways, among others), these organelles are gaining great relevance in addressing these questions [6]. More specifically, it has been shown that the mitochondrial DNA (mtDNA) of tumor cells accumulates many alterations, including punctual mutations, insertions, deletions and variations in mtDNA copy number (mtCNV), which trigger the metabolic reprogramming of transformed cells as a biological strategy that favors tumor cell proliferation and survival [7,8,9,10,11,12,13]. Even though the mutational spectrum of mtDNA has already been studied in different types of cancer, the mechanism by which mtDNA mutations contributes to tumor development is not yet well known [14,15,16]. Additionally, heteroplasmy, defined as the presence of two or more mtDNA variants coexisting within the same cell, and the proportion of mutated mtDNA (the heteroplasmy level) are crucial for the expression of specific pathological phenotypes [17,18,19]. Heteroplasmy has been proposed as an intermediate phase between the fixation or elimination of newly acquired mtDNA mutations. However, the critical threshold to observe a biochemical defect in the respiratory chain or for disease expression is undisclosed in cancer and scarcely analyzed in the studies that characterize the mtDNA variant landscape. Interestingly, many studies have shown an association among heteroplasmic levels and risk and survival of cancer [20,21,22,23,24]. Thus, this review focuses on describing the landscape of the heteroplasmy in cancer in order to contribute to the understanding of the biological processes involved, such as proliferation, metastasis and intratumoral heterogeneity, as well as heteroplasmy clinical implications.

## 2. Mitochondrial Genome

The mitochondrion is a semi-autonomous organelle whose main function is the synthesis of cellular adenosine triphosphate (ATP). However, mitochondria also play roles in metabolism regulation, cell death and signaling pathways involved in cell growth and proliferation [6]. The endosymbiotic theory indicates that the mitochondrion originated from endocytosis of an alpha-protobacterium by an ancestral pre-eukaryotic cell [6,25,26]. Throughout evolution, most of the mtDNA has migrated to the nucleus, leaving only a 16,569 base pair (bp) circular molecule [27]. This genome is constituted by two circular chains, a heavy strand (H) and a light strand (L) enriched in guanine and cytosine, respectively. mtDNA has 37 genes; two transcribe for ribosomal RNA (rRNA), 22 transcribe for transfer RNAs (tRNAs) and 13 transcribe for gene-encoding protein subunits of the enzyme complex of the oxidative phosphorylation system (OXPHOS) (Table 1). Additionally, the mitochondrial genome has a non-coding region of 1.1 kilobases (Kb), named D-Loop, which contains two hypervariable regions (HVR1 and HVR2) and sequences involved in the control of mtDNA replication and gene transcription [28,29]. The lack of histones, efficient DNA repair mechanisms and the proximity to reactive oxygen species (ROS) generated by the OXPHOS system (mainly from Complex I and III) explain the high mutation rate observed in mtDNA, which is between 10 and 17 times higher than that of the nuclear genome (nDNA) [28,29,30,31]. Furthermore, mtDNA is a multicopy genome, displaying variable copy numbers over time, among cell types, tissues, subjects, physiological conditions. The estimated copy number in mammalians ranges from 100 to 10,000 molecules per cell depending on the cell type and its energy requirement [32,33,34]. In humans, mtDNA is only inherited through the maternal line, and all alleles are passed down together as a single unit called haplotype, which could be shared in populations with a common ancestry. In fact, a set of haplotypes or haplogroup is useful for identifying ethnic groups or populations [35]. Studies suggest that specific haplogroups have conferred environmental adaptive advantages but are also associated with diseases such as cancer [36,37,38,39,40,41,42].

Even though mtDNA encodes only 13 of the approximately 1500 proteins that conform the mitochondria, its maintenance is essential for mitochondrial biogenesis [6,43]. The presence of alterations in mtDNA correlates with an inefficient OXPHOS system, decreased ATP synthesis and an increased ROS production rate, phenomena associated with pathological phenotypes [44,45,46,47,48].

## 3. Origins of Heteroplasmy and Its Impact on Disease

mtDNA replication, mediated by DNA polymerase γ (DNApolγ), is continuous and cell-cycle-independent. Replication is the primary source of new mutations in mtDNA. Despite the high fidelity of DNApolγ, the elevated replication rate and limited DNA repair mechanisms contribute to the maintenance of acquired mutations (point mutations, insertions, deletions, etc.) [10,49,50,51,52,53]. Due to the polyploid nature of mtDNA, mutations can be found in different proportions within a mitochondrion, cell, tissue or individual, giving rise to heteroplasmy [18,31,45,54,55]. Heteroplasmy can be higher if the mutated allele frequency (MAF) ranges from 20% to 95% or lower if the MAF ranges from 0.5% to 20%. A MAF <0.5% corresponds to a rare variant. If the allele represents more than 95% of the mtDNA molecules, this is considered homoplasmic [54,56].

**Figure 1:**
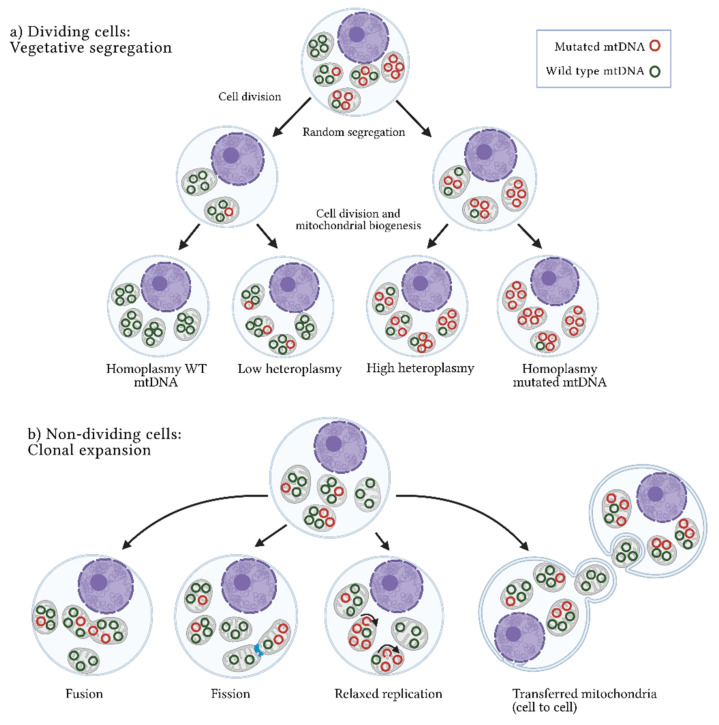
Heteroplasmy origin. Two mechanisms have been suggested to explain the origin and maintenance of the coexistence of wild-type and mutated mtDNA or heteroplasmy: (**a**) Vegetative segregation. In dividing cells, mitochondria are randomly distributed among daughter cells, resulting in different proportions of mutated mtDNA molecules between them. (**b**) Clonal expansion. In non-dividing cells, heteroplasmy is maintained through dynamic mitochondrial processes, such as fusion (union) or fission (separation) of mitochondria, relaxed replication (mtDNA replication independent of cell division) and intercellular mitochondrial transference. WT: wild-type; mtDNA: mitochondrial DNA molecules. Figure artwork created with BioRender.com.

**Figure 2:**
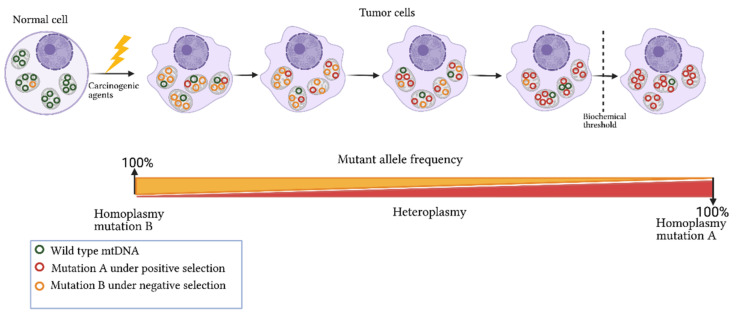
Dynamics of mitochondrial mutations in cancer. Exposure of a normal cell to carcinogenic agents leads to somatic mutation acquisition in mtDNA and changes in the germline mutation heteroplasmy. The frequency of acquired mutation can increase or decrease in tumor cells based on its nature and biological effect. For instance, if a mutation confers advantages to cell proliferation and survival, it will be selected and enriched in the tumor cell (mutation A). In contrast, a mutation compromising cell viability will be selected negatively (mutation B). Biochemical and phenotypic manifestation of the mtDNA mutations occurs only when a threshold level is exceeded. Figure artwork created with BioRender.com.

Heteroplasmy is maternally inherited, and its levels are determined during oogenesis. The proportion of mutated alleles differs between oocytes and then among offspring. Shifts in heteroplasmy levels have been observed in humans within a few generations, and a genetic bottleneck during the maternal transmission of mtDNA has been suggested to explain this phenomenon. The bottleneck could be derived from a reduction in the numbers of mtDNA molecules by three mechanisms: (a) passive reduction, where the mtDNA is destroyed through mitophagic processes; (b) mtDNA packaging into homoplasmic segregating units; and (c) focal replication, where specific populations of mtDNA are selected and replicated [57,58,59]. A bottleneck in oocytes, which divide rapidly after being fertilized, generates a heterogeneous distribution of mtDNA subpopulations in daughter cells [49,58,60,61]. Two main mechanisms by which heteroplasmy differs among cells have been described: vegetative segregation and clonal expansion. In the first case, changes in allelic frequencies occur through the mitochondria random distribution during mitosis; in the second case, present in post-mitotic cells or non-dividing cells, heteroplasmy depends on the mtDNA replication rate and other cellular processes such as mitochondrial fusion and fission, as well as mtDNA transference between cells (Figure 1) [49,57]. Thus, heteroplasmy is an extraordinarily complex and dynamic event that results from various stochastic and deterministic cellular processes [18,62,63,64,65,66]. Human pedigree analysis has shown that random genetic drift is involved in heteroplasmy and in combination with mitochondrial segregation, and the horizontal transfer of cell–cell mitochondria, modifies the allele frequencies of mtDNA over time. These frequencies are prone to increase or decrease depending on the effect of the variant [67]. In this context, heteroplasmy is a transitional event between the disappearance or fixation of alleles that alters the mitochondrial function or confers advantageous biochemical conditions that promote cell division (Figure 2) [57,68,69]. A scan of heteroplasmic sites in different tissues showed the allele-specificity and tissue-specificity of some variants, showing evidence of the presence of positive and negative selection that promote or inhibit particular conditions of mitochondrial function [70,71]. Transversions (point mutations that change a purine A or G by a pyrimidine T or C) and non-synonymous amino-acid changes were more commonly identified in a low heteroplasmic state, which reinforces the fact that, due to their potentially pathogenic nature, these changes are selected negatively to reduce the damage of protein subunits involved in mitochondrial function. In contrast, a positive selection of somatic heteroplasmic sites was observed both in liver tissue and in specific genes such as NADH:ubiquinone oxidoreductase core subunit 5 (*MT-ND5)*, suggesting that these mutations provide specific biochemical features to the tissue [70].

Heteroplasmy is a common phenomenon, and low levels of heteroplasmy are commonly found in healthy tissues; the presence of potentially pathogenic mutations is not always translated into changes in the cell function or in a specific phenotype [72,73,74]. The maintenance of normal mitochondrial function is explained by the existence of a “biochemical threshold”, a state where normal mtDNA buffers the damaging effect of mutated mtDNA molecules through the modulation of mitochondrial DNA replication, mitochondrial fission and fusion processes [73,75]. The threshold is tissue-specific, and the level of the mutated allele (heteroplasmy level) required to produce a pathological phenotype is dependent on the nature of the mutation [57,74,76]. Many studies have reported that heteroplasmy levels of mutations positively correlate with ROS production and cellular damage that is potentially expressed as a wide range of clinical manifestations [57,62,77]. For example, individuals carrying the A3243G mutation in heteroplasmy levels of 20%–30% commonly have type II diabetes mellitus; ranges from 50% to 80% of the mutated allele are manifested in myopathies, lactic acidosis or cardiomyopathies; in a homoplasmic state, A3243G carriers develop Leigh syndrome [78,79]. Additionally, the degree of heteroplasmy has been associated with different changes in the metabolic and epigenetic context [78].

## 4. Mitochondrial Alterations in Cancer

Cancer is a highly heterogeneous disease, and its etiology is usually multifactorial [80]. Since the description of the Warburg effect (aerobic glycolysis) and the recognition of metabolic reprogramming as a hallmark of cancer [81,82,83], mtDNA integrity has observed relevant in signaling pathways involved in normal and malignant cell proliferation, and thus tumor development and progression [15,21,44,84,85,86].

Studies based on mtDNA sequencing from paired tumor–normal tissue samples have indicated the spectrum of mtDNA alterations in different types of malignant entities [9,14,87], revealing that single nucleotide variants (SNVs) and alterations in the copy numbers of the entire mitochondrial genome, followed by deletions and insertions, are the most common mtDNA variation types involved in cancer [14,30,54]. Among the SNVs, G > A and T > C substitutions are the most frequent mutations (generated by biases in the replication process of the mtDNA) detected in cancer. These mutations are localized throughout the entire mtDNA and can be selected or eliminated along the tumor cell division [14,88]. Interestingly, differences in the mutated genes and in the mutation rate among malignancies have been observed. For example, *MT-ND5* is mutated in many types of cancer, NADH: ubiquinone oxidoreductase core subunit 4 (*MT-ND4)* is frequently mutated in lung and prostate cancer, and Cytochrome c oxidase I *(MT-CO1)* is mutated in breast tumors [14,88]. It is important to note that approximately 60%–80% of the mtDNA mutations correspond to non-synonymous amino acid changes [14,22,23,88]. One of the most studied mutations is A10398G in the NADH:ubiquinone oxidoreductase core subunit 3 (*MT-ND3)* gene, which results in a substitution of threonine by alanine in the encoded protein. Experimental data suggest that A10398G compromises the function of the ND3 complex I subunit of the OXPHOS system, and functional studies have shown that the G allele increases ROS production and promotes cell proliferation [89,90,91,92]. Even though numerous studies suggest that mtDNA mutations participate in cancer, the functional effect of most of them is still undeciphered and whether they play a role as drivers or passengers is debatable. To infer the role of mtDNA mutation in cancer development and progression or if they are under selection processes, the ratio of non-synonymous substitutions per non-synonymous sites (Ka) to the synonymous substitutions per synonymous sites (Ks) is estimated [22,93]. Assuming that the number of synonymous changes is more frequent than non-synonymous changes, Ka/Ks = 1 indicates neutral selection (reported in most types of cancer) and suggests an intact mitochondrial function in neoplastic cells and that the presence and levels of mtDNA mutations are derived from the tumorigenic environment [14]. Ka/Ks > 1 indicates positive selection and that mutation under this process confers biological advantages for tumor progression. When Ka/Ks < 1, it is hypothesized that mutations are selected negatively because of its deleterious effect on cell viability (Figure 2) [14,22]. Examples of positive and negative selection have been observed in breast and oral cancer [71,93] and in colorectal tumors [22,94], respectively.

Variation in the mtDNA copy number (mtCNV) is also commonly observed in cancer and has shown wide heterogeneity across different types of tumors and ethnicities. For instance, mtCNV is increased in ovarian tumors and colon cancer but decreased in myeloid leukemia, breast cancer, gastric cancer and hepatocellular carcinoma, among others [7,9,14]. Further, many studies have shown a correlation between mtCNV and the load of deleterious variants, which supports the theory that the genomic dose is modulated to compensate the erratic function of deleterious alleles [95,96]. Even though the biological role of these alterations in oncogenesis is still unknown, mtCNV has been associated with the risk of cancer and clinicopathological variables; indeed, mtCNV has been proposed as a potential prognostic biomarker in adrenocortical, kidney, pancreatic, thyroid, head and neck and colorectal tumors [9,97,98,99,100].

Somatic mtDNA insertions or deletions have also been implicated in cancer. As punctual mutations, the frequency of insertions and deletions varies among tumor types as documented in breast, colorectal, gastric and hepatic cancer [88,93,101,102,103]. For example, it has been reported that 45% of colorectal cancer patients are positive to insertions or deletions [93,101], but only 3% are positive in breast cancer [88]. The “common deletion” corresponds to a 4977-bp elimination between nucleotides 8470 and 13,447 of human mtDNA. This deletion has shown different frequencies among tumor types, ethnic origin [103] and noncancerous tissue versus cancerous tissues [102,103]. For example, Chen et al. reported that the mtDNA 4977-bp deletion was present in 6.73% of colorectal cancer tumors but was absent in their paired normal tissues [102].

Given the semi-autonomy of the mitochondria and its close interaction with the nuclear genome, a relationship between mitochondrial mutations and nuclear gene mutations to trigger carcinogenesis has been suggested [49,94,104]. In fact, a correlation between mitochondrial and nuclear mutational burdens has been observed in different types of cancer, being increased in renal and thyroid carcinomas [14]. Furthermore, the presence of deleterious mitochondrial mutations has been correlated with the overexpression of genes involved in altered signaling pathways in cancer, such as the TNFα pathway, OXPHOS and the protein secretion pathway [14]. Interestingly, a correlation of mitochondrial mutations with alterations in *FOXA1*, *MED12* and *MYC* nuclear genes was also reported, with a synergism between them in the clinical behavior of prostate cancer [23]. Even though evidence has shown that there is a mutational correlation between mtDNA and nDNA, it is important to note that approximately 20% of patients have mutations in mitochondrial genes but not in nuclear genes, suggesting that some of these mtDNA mutations are potentially responsible for carcinogenic development [14], but no studies characterizing the role of these alterations in cancer have been carried out.

The identification of all mtDNA alterations involved in cancer is a difficult task, and the results are often highly dependent on the technologies and approaches used. mtDNA mutation data can be obtained from off-target sequence analysis extracted from complete genomes and exome sequencing data, but the coverage and depth uniformity vary widely and could affect the variant-calling precision and the heteroplasmy determination. Further, molecular mechanisms under heteroplasmic or homoplasmic states contributing to the oncogenic process are still undeciphered [9,55,105,106].

## 5. Heteroplasmy Shifting in Cancer: Potentially Unique Profiles

Heteroplasmy of normal human cells is highly heterogeneous, with the frequency of heteroplasmic alleles differing even more among tissues. However, in cancer development, the dynamic of heteroplasmy seems to be an evolutionary process of mutational burden since tumor cells harbor additional homoplasmic and heteroplasmic mutations. Advances in next-generation sequencing (NGS) technologies have made it possible to explore (with high depth coverage) the spectrum of mitochondrial mutations and their heteroplasmy levels in paired tumor–normal tissues [14,22,71]. These studies have allowed researchers to identify both acquired mutations and shifting in heteroplasmy during cancer progression. In fact, dramatic shifts from the germline to tumor heteroplasmies have been documented in many types of cancer [17,23,71,88].

A pan-cancer study included 2658 paired tumor–normal tissue samples spanning 38 cancerous tissue types. In this study, approximately 85% of the substitutions were found with heteroplasmy levels <60% [14]. Heteroplasmy levels are widely heterogeneous between types of cancer and subjects. For example, heteroplasmy ranged from 2% to 49% in colorectal cancer, and heteroplasmy levels from 2% to 97.5% in oral and breast cancer. These findings revealed that heteroplasmy is tissue-specific, although it is not possible to discard the influence of selection processes (positive or negative) of the mutations based on the acquisition of favorable metabolic advantages for cell maintenance and proliferation (Figure 2) [17,88,93,94].

In mucosae-colorectal cancer samples, it was found that 90% of the tumors harbored at least one cancer-specific somatic mutation, and most of these mutations (>10%) were heteroplasmic. The authors observed that heteroplasmy changed among tissues and during tumor progression. A probable bottleneck process was suggested, based on the hypothesis that some heteroplasmic variants are eventually able to become dominant or lost in cancer cells based on their tumor-promoting effect. G1576C and G12009A mutations were the most enriched in tumor cells, as opposed to normal cells (7.8% in tumor versus <0.35% in normal cells, and 68.8% in tumor versus <0.35% in normal cells, respectively) [71].

Analyzing the spectrum of heteroplasmic sites in 100 colorectal tumors, Skonieczna et al. found that 76% of the colorectal tumors carried somatic mtDNA mutations randomly distributed along the entire mitochondrial genome. Overall, 43 heteroplasmic sites were detected in normal tissues, 22 of which were found as homoplasmic in tumors. For example, the minority variant at the T8610C position detected in normal tissue turned out to be the homoplasmic dominant variant in the corresponding cancer specimen, suggesting that these mutations could potentially be involved in the acquisition of advantages to ensure the survival of neoplastic cells [93].

In gingivobuccal oral squamous cell carcinoma, heteroplasmies ranged from 3% to 99%. Notably, nearly all non-synonymous somatic mutations were found to be heteroplasmic, while most synonymous and non-coding somatic mutations and germline mutations were found to be homoplasmic [21,94]. Given that the changes in coding regions presented a high pathogenicity score (>0.7), these authors suggested that the increased number of mutations in the heteroplasmy state could result in a dilution of the mutation with a pathogenic effect, trying to maintain mitochondrial function and favoring the adaptation of tumor cells and their eventual progression (Figure 2) [21,94]. In addition, in oral squamous cell carcinoma, extensive heteroplasmy was also found in the somatic mtDNA mutations. The heteroplasmy levels of some of these mutations were higher in malignant cells than in normal tissues, and those with a potential functional impact were increased in tumors [21].

Examples of heteroplasmy differences among paired tumor–normal tissues have also been described in breast cancer. McMahon and LaFramboise [22] found a total of 141 somatic mutations in a heteroplasmy state. In this study, it was suggested that heteroplasmy could be influenced by differences in the normal cell content or tumor heterogeneity rather than a heteroplasmic state within each tumor cell [22]. However, in previous work, we detected somatic mutations and germline variants displaying differences in the heteroplasmy levels among the normal and tumor tissues of breast cancer. For most germline variants, MAF was enriched in tumors in comparison with normal tissues, while for somatic mutations, MAF was extensively heterogeneous and 21.4% of somatic mutations were homoplasmic. Our study is similar to another that proposed a shift from germline heteroplasmy toward homoplasmy in tumor cells [88].

To determine the mitochondrial mutational landscape in prostate cancer, Hopkins et al. analyzed 384 patients and observed changes in the heteroplasmy levels of *MT-CO1* mutations, for instance, the 6419A allele, which was heteroplasmic within the normal samples but homoplasmic in the tumor [23].

**Figure 3:**
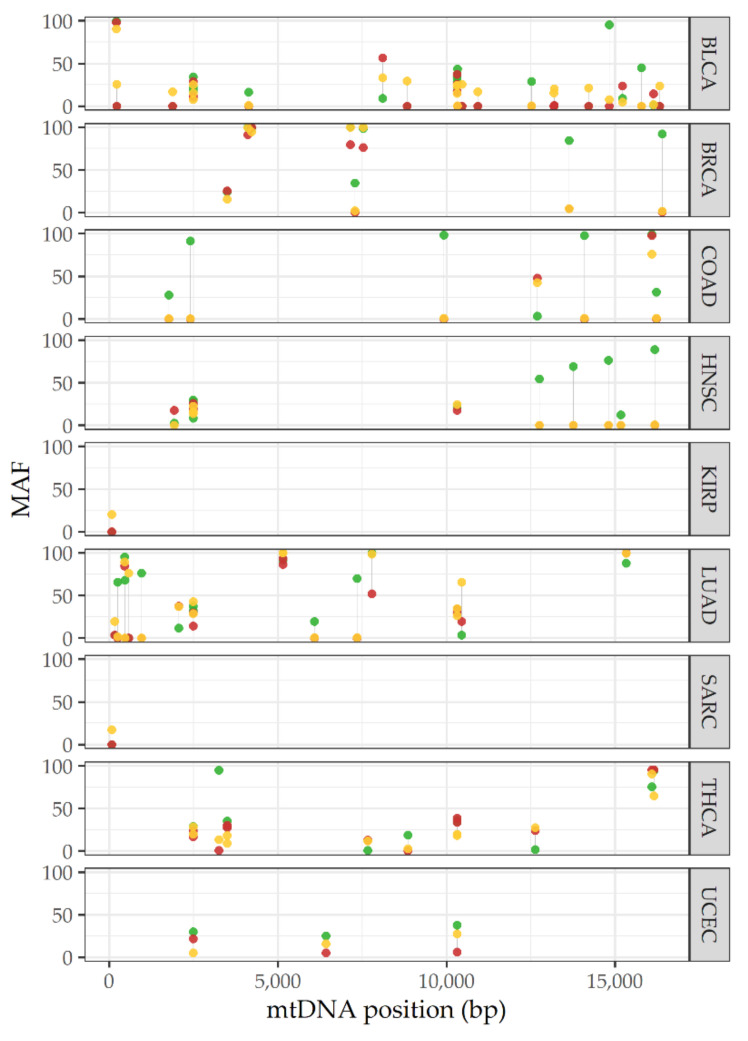
Landscape of heteroplasmy shifting in cancer. Mutant allele frequency (MAF) in peripheral blood (red points), adjacent tissue (yellow points) and tumor (green points) for each mitochondrial heteroplasmic site in several types of cancer. The graph is derived from 86 selected heteroplasmic sites reported by Grandhi et al. (public data) [17]. BLCA: bladder urothelial carcinoma; BRCA: breast invasive carcinoma; COAD: colon adenocarcinoma; HNSC: head and neck squamous cell carcinoma; KIRP: kidney renal papillary cell carcinoma; LUAD: lung adenocarcinoma; SARC: sarcoma; THCA: thyroid carcinoma; UCEC: uterine corpus endometrial carcinoma; bp: base pair.

In addition to the previous studies, Grandhi et al. recently published a robust heteroplasmy analysis on 24 types of cancer. These authors also described shifting heteroplasmy processes in cancer by analyzing 1916 whole genome sequences (WGS) from matched tumor-normal tissue samples (blood–tumor, adjacent tissue-tumor and blood-adjacent tissue-tumor). Extracting mtDNA data from WGS, the authors reported that tumor mitochondria have distinct genomic features and that tumor cells have a selective preference for mtDNA mutations and tolerate damaged mitochondrial genomes, which explains the heteroplasmy shifting observed in all studied cancer types [17]. Based on this study, we obtained the MAF from data reported on heteroplasmy sites of samples from blood-adjacent tissue-tumor matched tissues to visualize the heteroplasmy dynamic in each cancer type. In addition to the heteroplasmy shifting in tumor cells reported by Grandhi et al., we were able to observe a potential heteroplasmy profile specific to each type of cancer (Figure 3). Even though these observations provide data supporting the role of heteroplasmy in cancer progression, more studies are required.

The mechanisms involved heteroplasmy shifting in cancer have been rarely studied. However, there is evidence that cell identity and the nucleus-mitochondrial context modulate the energy performance of the OXPHOS system determining selection of specific mutant alleles [68]. For example, in renal carcinoma, it has been shown that the accumulation of fumarate leads to the inactivation of core factors responsible for mtDNA replication, altering the mitochondrial content [107]. Anomalies in mtCNVs may be generated in response to intrinsic and extrinsic stimuli, mtDNA mutations in the control region, altered expressions of mitochondrial transcription factor A (*TFAM*) and DNA polymerase gamma (*POLG*), mutations in nDNA-encoded genes involved in mitochondrial biogenesis and nuclear and mitochondrial epigenetic modifications [108,109,110]. For instance, the dysregulated expression of nuclear genes such as dynamin 1 such as (*DRP1*), mitofusin 1 (*MFN1*) and 2 (*MFN2*) mitochondrial fusion and fission proteins, BCL2 inter-acting protein 3 (*BNIP3*), PTEN-induced kinase 1 (*PINK1*) and hypoxia inducible factor 1 (*HIF1*), observed in lung, bladder and breast cancers, suggests increased mitochondrial dynamics [111,112]. The contribution of the tumor microenvironment in the modification of the allelic frequencies of mtDNA mutations was also proposed based on an analysis of primary tumors and their distant metastasis [15]. Moreover, bone marrow stromal cells have been reported to transfer functional mitochondria to acute myeloid leukemia blasts through NOX2-derived redox signaling [113,114]. Together, these mechanisms could play an important role in the development of an adaptive response to the tumorigenic environment, which is reflected in the modification of the allelic frequencies of mtDNA mutations.

Heteroplasmy could be influenced by the limitations of the used techniques to obtain the DNA (total DNA or mtDNA), sequencing platforms (depth, coverage), tissues (tumor cell heterogeneity and tumor cell enrichment), tissue origin, etc. Undoubtedly, the scanning of heteroplasmic sites in tumors accompanied by the analysis in paired-normal tissues has helped to identify a mutational dynamic of the mitochondria in cancer and to establish that this phenomenon contributes to cancer evolution. However, the heteroplasmy levels of the variants represent one of the main obstacles to decipher and clarify their participation in tumor development. To date, there is no experimental strategy that allows us to control the proportion of the mtDNA-mutated allele, but the search for heteroplasmic patterns in different types of cancer could increase the possibility of finding markers that improve diagnosis and allow for the prognosis of the patients.

Future studies exploring heteroplasmy should include the nuclear insertions of mitochondrial origin (NumtS), which have been implicated in several human diseases. NumtS are segments of the mtDNA that have been integrated into the nucleus along the evolutionary process and are estimated to occur at a rate of ∼5 × 10e^−6^ per germ cell per generation. Even though the presence of NumtS can mislead the identification of mtDNA mutations and the heteroplasmy evaluation, it has been found that NumtS are present in approximately 2% of tumors, reaching 5% in lung, skin, breast and uterine cancer and even 15% in HER2 subtypes of breast cancer and in squamous cell lung cancer [14]. These data expose the need to include NumtS as potential confounders in studies evaluating the role of heteroplasmy in cancer.

## 6. Tumor Microenvironment and Heteroplasmy

The tumor microenvironment influences several oncogenic processes such as immune evasion, tumor proliferation, metastasis and drug resistance [115,116,117]. These processes are modulated by tumor architecture and vasculature, interaction with normal and immune cells, cytokine release, altered cellular signaling and gene expression due to an acidic and hypoxic milieu, and the dysregulation of essential metabolites such as glucose and lactate [118,119,120,121,122,123]. For example, hepatocarcinoma cells cultured under hypoxic conditions (1% O_2_, 5 mM glucose, 10 mM lactate) showed an increase in the number of mutations in mtDNA and an overexpression of glycolytic enzymes such as hexokinase 2 (*HK2*) and glucose transporter (*GLUT*), in comparison with cells cultured under standard conditions (21% O_2_, 25 mM glucose) [116].

Even though evidence suggests that mitochondrial function is the center of metabolic plasticity of both tumor cells and cells in the environment, allowing their metabolism to be modulated under hypoxic conditions [117], the role of the microenvironment as a modifier of heteroplasmy in cancer has scarcely been explored. It is well known that hypoxia is a common phenomenon in solid malignant tumors [124]. Interestingly, experimental mice models revealed that the hypoxic condition modifies mtDNA and an increased oxygen concentration restricts the reduction in the mtDNA content and thus the MAF of mtDNA mutations [125]. In an in vitro model of early mammalian germ cell development from embryonic stem cells, Pezet et al. observed that differences in oxygen tension during early development modulate the amount of mtDNA and contribute to tissue-specific mutation loads [125]. Both inherited mutations and mitochondrial acquired mutations are selected based on environmental pressures and potentially confer characteristics that allow cancer cells to emerge and spread. It was hypothesized that heteroplasmic variability results from random genetic drift; however, data showing high differences in heteroplasmy levels in tissues of the same origin (mesodermal: bone marrow and lienal artery) and the same heteroplasmic frequency in tissues of different embryonic sources (endodermal: tonsils and mesodermal: joint cartilage) suggest that physiological and pathophysiologic biochemical conditions could modulate heteroplasmy [126,127].

Other recent studies have described the functional mitochondrial horizontal transfer from normal to tumor cells in acute myeloid leukemia, glioblastoma, osteosarcoma and breast and prostate tumor cells, probably as a mechanism for the restoration of mitochondrial basic functions, or for the adaptation to a high hypoxic and redox environment [113,114,128,129]. Undoubtedly, mitochondrial transfer could alter the heteroplasmy levels of mtDNA mutations in tumor cells.

In terms of the heteroplasmy and MAF in cancer, studies in paired samples from tumor-adjacent tissues have shown no statistically significant differences among tissues [130,131]. Moreover, Liu et al. [131], using data from Ling et al. [132] to trace the development and changes in mtDNA mutations in multiregional samples (26 locations) of the same tumor and non-cancer tissue, found that the MAF of heteroplasmies was similar in both tumor and nontumor tissues [131]. One interesting finding was that the non-synonymous heteroplasmy in tumors had a higher MAF than did normal tissues [131], a common phenomenon described in various tumor types [20,88]. In addition to inherited genetic factors, hormones, age, diet and exposure to carcinogens [130], the heteroplasmy of mitochondrial DNA mutations in cancer could be influenced by the microenvironment [133], but additional studies are needed to understand this relationship.

## 7. Heteroplasmy as a Potential Biomarker of Development and Progression in Cancer

The wide heterogeneity of mitochondrial alterations complicates both the elucidation of its functional effect in cancer as well as its association with clinical variables. Several studies have found the over-representation of groups of mutations associated with specific clinical features and prognosis, suggesting that these could be used as potential biomarkers [23,134,135].

For example, Kloss-Brandstätter et al. observed that patients with prostate cancer and a somatic mutation in mitochondrial tRNAs have significantly higher prostate specific antigen (PSA) values at diagnosis than patients without a somatic tRNA mutation (14.25 ± 5.44 versus 7.15 ± 4.32 ng/mL; *p* = 0.004). Furthermore, differences in the proportion of specific mutations between benign and cancerous tissues (prostate tumor and seminal vesicle tumor) were observed, supporting the hypothesis that the proportion of mutated mtDNA molecules increases with malignancy [24]. In addition, Hopkins et al. observed that patients with mutations in the hypervariable region 1 (HVR1) had a longer survival compared to those with mutations in the origin of H-strand replication (OHR) that had a shorter survival time. These results suggest that a mutational analysis focused on functional regions of mtDNA could be relevant in the clinical assessment of patients with cancer. For instance, mutations in *MT-CO2*, *MT-CO3*, *MT-ATP8* and *MT-ND4L* genes, HVR1, OHR and conserved sequence block 1 (CSB1) regions could be useful for risk classification in prostate cancer. Those who carried mutations in *MT-CO2*, *MT-CO3* and HVR1 were classified as low-risk, and patients bearing mutations in *MT-ATP8*, OHR, *MT-ND4L* and CSB1 were classified as high-risk [23]. On the other hand, in oral carcinoma, an over-representation of non-synonymous mutations was found in patients with metastases to lymph nodes; the absence of somatic mutations was correlated with a better prognosis [94]. The potential implications of heteroplasmic patterns in cancer has rarely been explored. Several studies have reported an association among heteroplasmic levels with demographic and clinical variables such as age, time of diagnosis, tumor activity and survival [23,24,92,135].

African American breast cancer patients showed statistically significant high heteroplasmic changes with an over-representation of the mutated allele in the tumor compared to Caucasian patients. Furthermore, patients with HER2-positive tumors showed higher heteroplasmy levels compared to HER2-negative ones, suggesting that heteroplasmy may reflect the aggressiveness of the breast cancer subtypes [22].

In prostate cancer, a relationship between tumor activity and the heteroplasmy levels of several mutations has also been reported; when the level and degree of heteroplasmy increased, so did the tumor activity [24]. On the other hand, the presence of at least one mutation in more than 20% of heteroplasmy level in primary oral tumors was significantly associated with a lower survival in patients [21]. In lung cancer, the A10398G variant was found in a heteroplasmic range from 0.31% to 97.04%, the patients with high levels of heteroplasmy being those who showed a greater survival than positive subjects with low heteroplasmy levels [20].

In addition to the above-referred studies suggesting a relationship among heteroplasmy and cancer severity and progression, functional studies have demonstrated the impact of mtDNA mutations on tumor proliferation and metastasis development [136,137]. Ishikawa et al., using transmitochondrial hybrid models from lung cell lines, established that the G13997A mutation in the *MT-ND6* gene alters the Complex I function of the OXPHOS system, promoting the overproduction of ROS, which activates hypoxic signaling pathways involved in the metastasis process [137]. Considering the heteroplasmy phenomenon, other authors have compared the MAF of mtDNA mutations identified in primary breast, colorectal and prostate tumors with their respective metastases [15,133,138]. These studies documented that several mutations detected as heteroplasmic in the primary tumor were found in a homoplasmic state in their metastases [15,133]. For example, the G9820A mutation (*MT-CO3* gene) was heteroplasmic in primary prostate tumors and soft tissue metastasis but was homoplasmic in bone metastasis. These authors also detected negatively selected mutations that were within the soft tissue metastatic site, including a mutation at nucleotide position 9377 in *MT-CO3* [133] and T1924C and T2305C mutations (*MT-RNR1* gene) identified in colorectal tumors, where the MAF increased in metastases (MAFs of 0.7% versus 42% and of 9% versus 34%, respectively) [138]. Additionally, in head and neck cancer, tumor-specific mutations were identified in different regions of adjacent normal tissue, metastatic lymph nodes, sputum and serum from patients with a heteroplasmy of up to 1%, suggesting that mitochondrial mutations can detail the evolution of tumor development and can be used to monitor the progression of neck cancer [106]. These findings together suggest that a tissue-specific selection process for mtDNA mutations confers advantages for cancer metastasis and that heteroplasmy could have potential use as a progression biomarker in human malignancies [15,133].

Even though functional studies that explain the impact of heteroplasmy on survival are lacking, it has been proposed that the accumulation of mutations leads to alterations in mitochondrial function that leads to cell death and that changes in heteroplasmy levels could be a way of modulating sensitivity to anticancer treatments [20,139]. For these reasons, studies focused on multiregional sequencing of the tumor microenvironment will allow us to determine the impact of mitochondrial genomics on the biology and clinical development of malignant entities.

## 8. Heteroplasmy in Epigenetic Modulation

Even though mtDNA mutations are found in more than 90% of individuals with mitochondrial diseases, only 10%–40% of them develop severe clinical manifestations. The heterogeneity in the penetrance of the mitochondrial mutations suggests the presence of additional factors for developing a pathological phenotype. These factors include heteroplasmy levels, a nuclear genetic background, an interaction among mitochondrial and nuclear mutations, and environmental components that can modulate the epigenetic response and could contribute to mtDNA mutation penetrance [140].

The presence of direct epigenetic modifications in the mtDNA molecule is disputed [49]. However, evidence of the presence of 5-methylcytosine (5mC) and 5-hydrxymethylcytosine (5hmC) that are located mainly in non-CpG sites and are enriched in the L-strand, specifically in the origin of L-strand replication (OLR) and in tRNAs, suggests the presence of epigenetic mechanisms influencing the regulation of mtDNA replication and expression processes [140,141,142]. Similarly, isoforms of DNA methyltransferases (DNMT1, DNAMT3A and DNAMT3B) have been identified in the mitochondrial matrix, which binds to mtDNA and modulates its global methylation [140,143]. DNMT expression is regulated by nuclear transcription factors, such as nuclear respiratory factor 1 (NFR1) and peroxisome proliferator-activated receptor γ-coactivator 1α (PGC1α). These factors are expressed under hypoxic conditions, which shows evidence of the strong crosstalk between the nuclear response to changes in the cellular environment and the regulation of mitochondrial expression [144].

mtDNA methylation patterns are modified by oxidative damage, which decreases as ROS concentration increases, suggesting the presence of mechanisms that compensate cell damage through the activation of mtDNA transcription processes and mitochondrial biogenesis [8,140]. This idea is reinforced by data showing that, while the mtDNA content decreases, the methylation rate of the D-Loop region increases [145]. Otherwise, differentially methylated regions have shown a correlation with biological and clinical features. For example, reduced methylation in the *MT-CO2* gene has been proposed as a marker of senescence in heart mesenchymal stem cells [146], as well as the hypomethylation of the D-Loop region in colorectal tumors correlated with the increased expression of the *MT-ND2* gene and with clinicopathological data [147]. On the other hand, evidence proposed that mitochondria transmit information regarding their functional status to the nucleus through signaling pathways in order to trigger a cellular adaption response to the environment [148]. This retrograde signaling has been suggested, as orchestrated by modifications at the epigenetic level in the nDNA [148,149].

**Figure 4:**
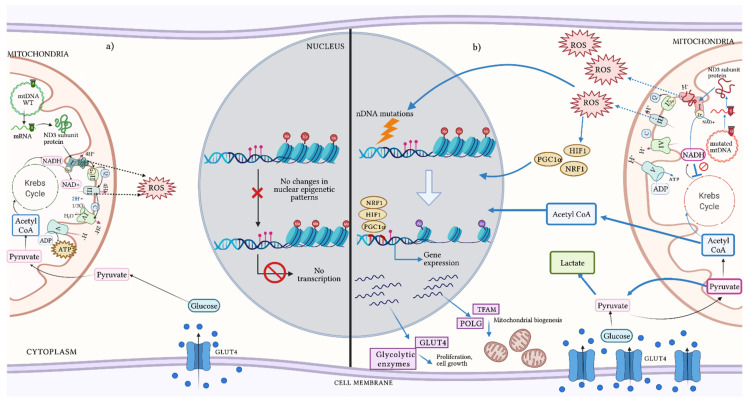
Crosstalk between mtDNA and nDNA. mtDNA codes for the most hydrophobic subunits of the OXPHOS system and the assembly of the complexes assembly is carried out in a perfectly coordinated manner between mtDNA and nDNA. (**a**) The appropriate function of the OXPHOS system maintains an efficient electron transfer and ATP synthesis that reduce NADH generated during glucose metabolism. (**b**) The presence of mtDNA mutations in coding regions can lead to the structural or functional alteration of protein subunits, resulting in an inefficient OXPHOS system and thus a high production of ROS. Dysfunction of the OXPHOS system inhibits the Krebs cycle and modifies the concentrations of important metabolites such as pyruvate and acetyl-CoA. These molecules alter the histone acetylation and mtDNA methylation levels. High concentrations of pyruvate, acetyl-CoA and ROS increase the acquisition of nDNA mutations and favor a hypoxic state that activates transcription factors such as HIF1, NRF1 and PGCa. These proteins increase the expression of genes involved in glycolytic metabolism and mitochondrial biogenesis processes, which have been associated with increased tumor growth and malignant cell proliferation. ADP: adenosine diphosphate; ATP: adenosine triphosphate; GLUT4: glucose transporter type 4; HIF1: hypoxia inducible factor 1; mRNA: messenger RNA; mtDNA: mitochondrial DNA; NAD+: nicotinamide adenine dinucleotide (oxidated form); NADH: nicotinamide adenine dinucleotide (reduced form); nDNA: nuclear DNA; NRF1: nuclear respiratory factor 1; OXPHOS: oxidative phosphorylation; PGC1α: proliferator-activated receptor γ-coactivator 1α; POLG: DNA polymerase gamma; ROS: reactive oxygen species; TFAM: mitochondrial transcription factor A; WT: wild-type. Figure artwork created with BioRender.com.

The participation of mtDNA in nDNA epigenetic modulation was observed in tumor cells lacking mtDNA. These cells presented different nuclear methylation sites versus their counterpart with mtDNA [148]. Changes in nuclear epigenetic patterns have also been observed through the variation of available metabolites, such as acetyl-CoA, α-ketoglutarate, 2-hydroxyglutarate and S-adenosyl-methionine (SAM), which are fundamental substrates in DNA methylation reactions [141]. In fact, variations in 2-hydroxyglutarate levels have been correlated with the dysfunction of Complexes I and III, causing an altered redox state modifying the methylation landscape of nDNA (Figure 4) [148].

Additional evidence supporting the role of the mitochondrial genetic background in nDNA methylation patterns is derived from the study of haplogroups. Individuals carrying the J haplogroup present higher levels of methylation than carriers of the H, U, X and T haplogroups. This could be explained by the modulation of SAM levels and the correlation with the overexpression of methionine adenosyl transferase 1A (MAT1A) [141,143]. However, the complexity of the mutational landscape of mtDNA is elevated after heteroplasmy level involvement, which is modified by environmental factors such as the oxygen level. Regarding this, the heteroplasmy of the T3395C variant is low in individuals living in low-altitude areas but can reach a heteroplasmy of up to 25% in people from Tibet (14,370 feet) [49].

Recent studies have shown that variations in the percentage of mutated mtDNA molecules generate changes in the transcriptomic profiles of mtDNA, which, through the modulation of intermediate metabolites, impact gene expression and the epigenomic patterns of nDNA [78,141,150,151]. Thus, the different levels of heteroplasmy can promote changes that correlate with normal or pathological phenotypes [150,152]. For example, the degree of dysfunction in mitochondrial protein synthesis generated by the different levels of heteroplasmy of the A3243G mutation modulates the concentration of acetyl-CoA and α-ketoglutarate. These molecules induce a cellular state with an altered redox environment that eventually modifies the histone acetylation and methylation patterns [78].

Research describing the landscape of mitochondrial mutations in cancer remains inconclusive to date. The methodological strategy used could partially explain this issue, but it is important to consider that a mutation in mtDNA may not be the only cause of the oncogenic process by itself; it may also be the sum of its heteroplasmic levels and its interaction with other mitochondrial and nDNA mutations. All these factors together might potentiate the pathological effect of a specific mtDNA mutation inducing epigenetic reprogramming and influencing tumor development and progression (Figure 4) [149].

## 9. Heteroplasmy Shifting as Therapeutic Strategy

There are currently strategies based on mitochondrial gene editing that have been proposed as a therapeutic option for restoring the OXPHOS system in diseases caused by mtDNA mutations [153]. However, the current mitochondrial disease treatments are palliative and are focused on reducing the damage caused by ATP deficiencies and ROS production. For example, the administration of antioxidants prevents oxidative stress, and ketogenic diets stimulate mitochondrial biogenesis via beta-oxidation activation [62,154,155].

Strategies such as mitochondrial transplantation have also been considered and focus on the prevention of a mutated mtDNA transmission from the germline. In this methodology, the nuclear genetic material of the pre- or post-fertilized oocyte is isolated and inserted into an anucleated oocyte with normal mitochondria obtained from a healthy donor [62]. Even though using this approach has shown a restoration of the OXPHOS system function, a subsequent reversal of the effects has also been detected, possibly caused by contamination of the extracted nucleus with mutated mtDNA. In addition to the complexity of these methods, the ethical issues surrounding embryo manipulation confers difficulties in clinical application as an optional therapy tool [62].

**Figure 5:**
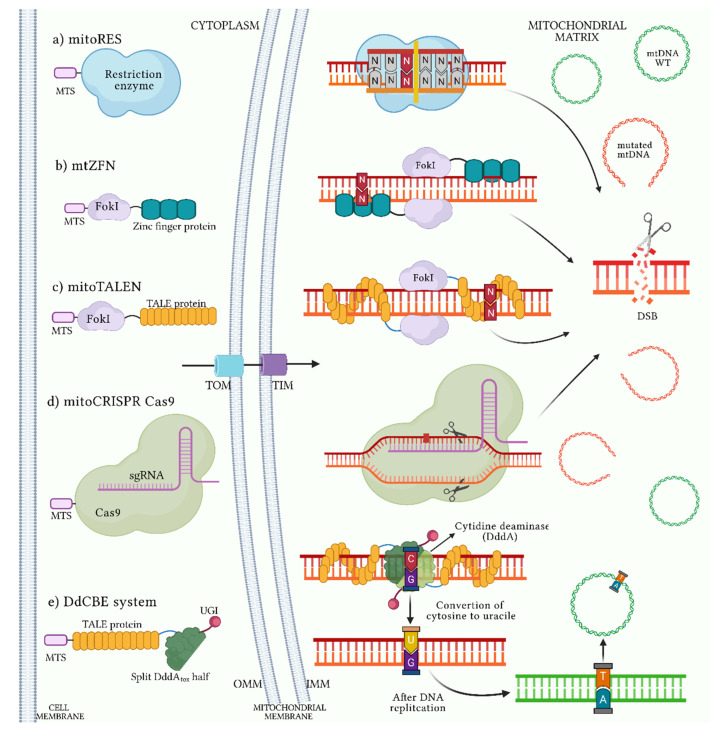
Heteroplasmic shifting strategies. The biological systems for modifying heteroplasmic levels of mtDNA mutations are presented. (**a**) mitoREs recognize specific sequences originated by acquired mtDNA mutations. (**b**) mtZFNs and (**c**) mitoTALENs can expand the recognition sequences for mtDNA mutations, resulting in more specificity. (**d**) The mitoCRISPR Cas9 system uses sgRNA for the mutation recognition. mitoREs, mitoZFNs, mitoTALENs and CRISPRCas9 systems use their endonuclease activity to induce DSB, linearizing and degradation of mutated mtDNA. (**e**) The DdCBE system can mediate the C to U conversion of C–G base pairs. Given its dependence on mtDNA replication, the newly synthesized mtDNA molecule will have the T–A base pairs, thus modifying the levels of heteroplasmy. DdCBE: DddA-derived cytosine base editor; DSB: doble-strand break; IMM: inner mitochondrial membrane; mitoCRIPR/Cas9: mitochondrial clustered regularly interspaced short palindromic repeats/CRISPR associated protein 9; mitoREs: mitochondrial restriction endonucleases; mitoTALEN: mitochondrial transcription activator-type effector endonucleases; mitoZFN: mitochondrial zinc finger coupled endonucleases; MTS: mitochondrial target sequence; OMM: outer mitochondrial membrane; sgRNA: guide RNA; TIM: translocase of the inner membrane; TOM: translocase of the outer membrane; UGI: uracil glycosylase inhibitor. WT: wild-type. Figure artwork created with BioRender.com.

Strategies focused on changing the proportion of mutated mtDNA molecules have been suggested as therapeutic options for individuals carrying somatic mutations and a dysfunctional OXPHOS system (Figure 5) [153,154,156,157,158]. One strategy is employing mitochondrial restriction endonucleases (mitoREs) to selectively induce a double-stranded break (DSB) in the mutated mtDNA (Figure 5a) [159,160]. Since mitochondria do not have repair mechanisms to mitigate this type of damage, linearized DNA is degraded, leaving normal mtDNA molecules in a near-homoplasmic state [62,161,162]. The main disadvantage of mitoREs is that not all mutations generate a restriction site to endonucleases. To overcome this, zinc finger-coupled endonucleases (ZFNs) [163,164,165,166] and transcription-activator-type effector endonucleases (TALENs) have been developed (Figure 5b,c) [167,168,169,170]. These systems possess modifiable DNA-binding domains that are associated with mutated mtDNA that induces a DSB of mtDNA and its degradation. ZFNs and TALENs are more specific than mitoREs, and a shift in heteroplasmy towards normal mtDNA has been demonstrated in cell lines and animal models using both methods [171,172]. However, the presence of off-target effects, the packaging limitations of molecular systems toward mitochondria and immunogenicity are some of the challenges that make clinical applications of ZNF and TALEN strategies difficult [153,173].

Recently, the clustered regularly interspaced short palindromic repeats/CRISPR associated protein 9 (CRISPR/Cas9) edition system has been explored to modulate heteroplasmy (Figure 5d) [174]. This system is based on the use of guide RNA (gRNA) that targets the mutation, but controversial results and the insurance of a delivery system for gRNA into mitochondria are the main unsolved obstacles. In addition, the existence of specific mitochondrial mechanisms that allow for the transport of RNA into the mitochondrial matrix is unknown [175].

So far, the manipulation of mtDNA has been based solely on the degradation of the mutated molecule, which can be detrimental to cells when the mutation is in a homoplasmic state. Recently, a mitochondrial editing system called DddA-derived cytosine base editor (DdCBE) has been reported as a modifier of the C–G base pairs to T–A. The DdCBE complex is made up of inactive halves of a bacterial toxin with cytidine deaminase (DddA) activity, TALE proteins that bind to specific mtDNA sequences, an uracil glycosylase inhibitor (UGI) protein and a mitochondrial recognition sequence (Figure 5e) [176]. Since DdCBE is a system that depends on the subsequent replication of mtDNA, the maximum efficiency of base conversion is 50%. Considering that most mutations have a pathogenic effect, reaching 80% heteroplasmy [58,73], this approach would reduce the heteroplasmy percentage of pathogenic mutations to a tolerable threshold for the cell [176].

These tools seem promising for the treatment of diseases caused by mtDNA mutations. In fact, by using mtZFNs to eliminate select mutant mtDNA molecules, it was possible to enrich the wild-type mtDNA proportion and rescue heteroplasmic cells from pathological phenotypes [165]. Gammage et al. demonstrated the utility of mtZFN-based approaches to rescue cells carrying the G14459A mutation (*MT-ND6* gene) and the “common deletion” associated with Leber’s hereditary optic neuropathy plus dystonia. In the same way, a shifting of heteroplasmy levels from T8993G (*MT-ATP6* gene) mutation, which is associated with neuropathy, ataxia and retinitis pigmentosa, was induced to restore the wild-type allele proportion (up to 90%) and the normal phenotype [165,169,177]. It has been proposed that factors involved in mitochondrial biogenesis and metabolism in cancer are a potential therapeutic target [178,179,180]; however, the application of tools focused on the reduction of the proportion of mutant DNA alleles has not been explored. The complexity of human malignancies, in addition to the lack of identified driver mtDNA mutations and their pathogenic heteroplasmy thresholds, makes the use of these techniques to shift heteroplasmy in tumor cells difficult.

## 10. Conclusions

The study of mitochondrial genome alterations in cancer is becoming increasingly relevant because of their potential role in cell proliferation and metastasis and clinical implications. Indeed, numerous studies propose that specific mutations, mutational burdens and heteroplasmy levels could be used as biomarkers to predict the risk of developing cancer, to make diagnoses and prognoses and to identify patients of several types of cancer that are at high risk of relapsing. Regardless the available data evidencing correlations among heteroplasmy and cancer-related phenotypes, whether the heteroplasmy or the variability in mtDNA copy number in cancer are cause or consequence is still unknown. However, determining the functional effect of mtDNA mutations in cancer is a difficult task, more so if heteroplasmy levels are considered. Thus, at this time, we are far from using heteroplasmy as a biomarker in the oncology clinical field. Integrative analyses including genomics, transcriptomics and epigenomics, of both mitochondrial and nuclear genomes, are necessary to clarify the participation of mutated mtDNA in the biology and clinical behavior of cancer.

## Figures and Tables

**Table 1:** Encoded mitochondrial DNA genes.

Gene Symbol	Name	Function	OXPHOS Complex	Nucleotide Start Position	Nucleotide End Position
*MT-ATP6*	ATP synthase membrane subunit 6	Proton transmembrane transporter activity	V	8527	9207
*MT-ATP8*	ATP synthase membrane subunit 8	Proton transmembrane transporter activity		8366	8572
*MT-CO1*	Cytochrome c oxidase I	Electron transport Heme binding		5904	7445
*MT-CO2*	Cytochrome c oxidase II	Electron transport	IV	7586	8269
*MT-CO3*	Cytochrome c oxidase III	Electron transport		9207	9990
*MT-CYB*	Cytochrome b	Electron transport Heme binding	III	14747	15887
*MT-ND1*	NADH: ubiquinone oxidoreductase core subunit 1	Proton pumping Reduction site for ubiquinone		3307	4262
*MT-ND2*	NADH: ubiquinone oxidoreductase core subunit 2	Proton pumping		4470	5511
*MT-ND3*	NADH: ubiquinone oxidoreductase core subunit 3	Proton pumping	I	10059	10404
*MT-ND4*	NADH: ubiquinone oxidoreductase core subunit 4	Proton pumping		10760	12137
*MT-ND4L*	NADH: ubiquinone oxidoreductase core subunit 4L	Proton pumping		10470	10766
*MT-ND5*	NADH:ubiquinone oxidoreductase core subunit 5	Proton pumping		12337	14148
*MT-ND6*	NADH:ubiquinone oxidoreductase core subunit 6	Proton pumping		14149	14673
*MT-RNR1*	12S rRNA	12S ribosomal RNA Small subunit	-	648	1601
*MT-RNR2*	16S rRNA	16S ribosomal RNA Large subunit	-	1671	3229
*MT-TA*	tRNA-Ala	tRNA for alanine	-	5587	5655
*MT-TC*	tRNA-Cys	tRNA for cysteine	-	5761	5826
*MT-TD*	tRNA-Asp	tRNA for aspartic acid	-	7518	7585
*MT-TE*	tRNA-Glu	tRNA for glutamic acid	-	14674	14742
*MT-TF*	tRNA-Phe	tRNA for phenylalanine	-	577	647
*MT-TG*	tRNA-Gly	tRNA for glycine	-	9991	10058
*MT-TH*	tRNA-His	tRNA for histidine	-	12138	12206
*MT-TI*	tRNA-Ile	tRNA for isoleucine	-	4263	4331
*MT-TK*	tRNA-Lys	tRNA for lysine	-	8295	8364
*MT-TL1*	tRNA-Leu (UUA/G) 1	tRNA for leucine 1	-	3230	3304
*MT-TL2*	tRNA-Leu (CUN) 2	tRNA for leucine 2	-	12266	12336
*MT-TM*	tRNA-Met	tRNA for methionine	-	4402	4469
*MT-TN*	tRNA-Asn	tRNA for asparagine	-	5657	5729
*MT-TP*	tRNA-Pro	tRNA for proline	-	15956	16023
*MT-TQ*	tRNA-Gln	tRNA for glutamine	-	4329	4400
*MT-TR*	tRNA-Arg	tRNA for arginine	-	10405	10469
*MT-TS1*	tRNA-Ser (UCN) 1	tRNA for serine 1	-	7446	7514
*MT-TS2*	tRNA-Ser (AGU/C) 2	tRNA for serine 2	-	12207	12265
*MT-TT*	tRNA-Thr	tRNA for threonine	-	15888	15953
*MT-TV*	tRNA-Val	tRNA for valine	-	1602	1670
*MT-TW*	tRNA-Trp	tRNA for tryptophan	-	5512	5579
*MT-TY*	tRNA-Tyr	tRNA for tyrosine	-	5826	5891

ATP: adenosine triphosphate; NADH: nicotinamide adenine dinucleotide reduced; OXPHOS: oxidative phosphorylation system, I: NADH:ubiquinone oxidoreductase complex; III: cytochrome bc1 complex; IV: Cytochrome c Oxidase complex; V: Mitochondrial ATP synthase complex; -: no OXPHOS complex.

## Data Availability

Not applicable.
